# Predominant neurological phenotype in a Hungarian family with two novel mutations in the *XPA* gene—case series

**DOI:** 10.1007/s10072-019-04044-6

**Published:** 2019-09-02

**Authors:** Dénes Zádori, László Szpisjak, István Balázs Németh, Zita Reisz, Gabor G. Kovacs, Noémi Szépfalusi, Viola Luca Németh, Zoltán Maróti, Edit Tóth-Molnár, Judit Oláh, László Vécsei, Péter Klivényi, Tibor Kalmár

**Affiliations:** 1 Department of Neurology, Faculty of Medicine, Albert Szent-Györgyi Clinical Center, University of Szeged, Szeged, Hungary; 2 Department of Dermatology and Allergology, Faculty of Medicine, Albert Szent-Györgyi Clinical Center, University of Szeged, Szeged, Hungary; 3 Department of Pathology, Faculty of Medicine, Albert Szent-Györgyi Clinical Center, University of Szeged, Szeged, Hungary; 4 Institute of Neurology, Medical University of Vienna, Vienna, Austria; 5 Genetic Diagnostic Laboratory, Department of Pediatrics and Pediatric Health Center, University of Szeged, Szeged, Korányi fasor 14-15, Szeged, H-6720 Hungary; 6 Department of Ophthalmology, Faculty of Medicine, Albert Szent-Györgyi Clinical Center, University of Szeged, Szeged, Hungary; 7 MTA-SZTE Neuroscience Research Group, University of Szeged, Szeged, Hungary; 8 Interdisciplinary Excellence Center, University of Szeged, Szeged, Hungary

**Keywords:** Xeroderma pigmentosum group A, Neurological, Ataxia, Parkinsonism, Cognitive, Neuropathology

## Abstract

**Objective:**

The prevalence of xeroderma pigmentosum (XP) is quite low in Europe, which may result in a delay in determining the appropriate diagnosis. Furthermore, some subtypes of XP, including XPA, may manifest themselves with quite severe neurological symptoms in addition to the characteristic dermatological lesions. Accordingly, the aim of the current study is to highlight the predominant neurological aspects of XPA, as well as mild-to-moderate dermatological signs in a Hungarian family with 5 affected siblings.

**Case reports:**

The symptoms of the Caucasian male proband started to develop at 13–14 years of age with predominantly cerebellar, hippocampal, and brainstem alterations. His elder sister and three younger brothers all presented similar, but less expressed neurological signs. The diagnostic work-up, including clinical exome sequencing, revealed 2 novel compound heterozygous mutations (p.Gln146_Tyr148delinsHis, p.Arg258TyrfsTer5) in the *XPA* gene. Surprisingly, only mild-to-moderate dermatological alterations were observed, and less severe characteristic ophthalmological and auditory signs were detected.

**Conclusions:**

In summary, we present the first family with genetically confirmed XPA in the Central-Eastern region of Europe, clearly supporting the notion that disturbed function of the C-terminal region of the XPA protein contributes to the development of age-dependent neurologically predominant signs. This case series may help clinicians recognize this rare disorder.

**Electronic supplementary material:**

The online version of this article (10.1007/s10072-019-04044-6) contains supplementary material, which is available to authorized users.

## Introduction

Xeroderma pigmentosum (XP) is a rare autosomal recessively inherited condition with 100% penetrance [1] and only with a prevalence of 2.3 per million in Western Europe [2]. There are several distinct clinical subtypes of XP, including the variant form and those marked with letters A–G based on the different genetic cause. The characteristic cutaneous signs are exaggerated sensitivity to sunlight, early development of freckle-like lentiginous pigmentation, and increased propensity for the formation of malignant skin tumors. The ocular alterations may involve conjunctival xerosis, corneal drying, and conjunctivitis. Neurological signs, such as intellectual disability, speech disturbance, sensorineural hearing loss, peripheral neuropathy, corticospinal alterations, and movement disorders with a predominant cerebellar ataxia resulting in severe walking disability, may also develop, especially in XPA [1, 3]. The aim of the current study is to present a family with 5 affected siblings diagnosed with novel compound heterozygous mutations in the XPA gene in accordance with the CARE (CAse REport) guidelines [4]. Additionally, this report is unique in its detailed genetical, neuropathological, ophthalmological, and dermatopathological assessment in addition to the comprehensive delineation of clinical symptoms and signs. Accordingly, this case series may add to the known phenotypic spectrum of XPA.

## Case reports

### Case histories, neurological, and related alterations

**Fig. 1:**
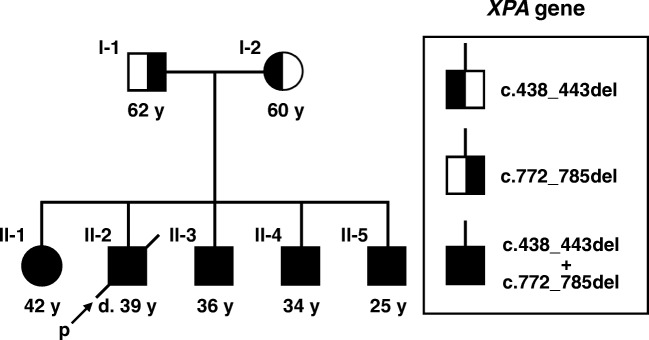
Pedigree of the assessed family with mutation in the *XPA* gene

The Caucasian male proband (II-2 in Fig. 1), who died at 39 years of age, was first admitted to our clinic for a diagnostic work-up of his unknown cognitive and movement disorder at 36 years of age. In addition to the presence of a slightly exaggerated sunburn reaction, his neurological symptoms started to develop at his 13–14 years of age. His speech became slurred and his cognitive functions deteriorated as well, resulting in progressive and severe learning disabilities. He completed 11 classes and later he worked in a twine factory until his 26 years of age, and then his disability led to retirement, and he became dependent on his parents. Repeated falls occurred with scarring and his swallowing functions and vision deteriorated as well.

Upon neurological examination, he presented signs of disturbed eye movements (exophoria, restricted eye movements in all directions with diplopia, gaze-evoked nystagmus), dysarthria, hypo-/areflexia, pathological reflexes, and decreased sense of vibration. Furthermore, movement disorder with dominating ataxia and parkinsonism (bilateral dysmetria, cerebellar predominant mixed limb ataxia more pronounced in the legs, truncal ataxia, severe postural instability, broad-based, ataxic gait, moderate, mainly left-sided bradykinesia and upper limb dystonia, mild rigidity on provocation, mild postural tremor, and occasional myoclonic jerks) was also detected (Supplementary Material 1.1 and 2). The myoclonic jerks were spontaneous, distal, and as they can be exacerbated by sensory stimuli, they were presumed to be cortical. The neuropsychological assessment revealed the signs of severe cognitive dysfunction confined to two functional neuroanatomical networks, the hippocampus-dependent and that related to the prefronto-cerebellar system, with similar degrees of impairment (Supplementary Material 1.2).


ESM 2(AVI 13048 kb)


The brain MRI revealed pronounced generalized atrophy with slight predominance regarding the parieto-occipital and cerebellar structures (Supplementary Material 1.3). The degree of atrophy seemed to correlate with the severity of neurological signs.

Electroneurography demonstrated mixed sensorimotor lower limb predominant polyneuropathy.

Following his death caused by aspiration pneumonia resulting from dysphagia as a part of progressive neurological impairments, comprehensive post mortem neuropathological assessment was carried out. The most prominent alterations demonstrated by that were asymmetrical hippocampal sclerosis and Purkinje cell degeneration along with moderate loss of neurons in the substantia nigra and a scattered infiltration of CD8-positive T lymphocytes (for details, please see Supplementary Material 1.4 and 1.5).

Regarding the family history of the proband, the presence of similar, but less expressed, deterioration was identified in his sister (II-1, 3 years older than the proband—Supplementary Materials 1.6 and 3), and his three brothers (II-3, 3 years younger than the proband—Supplementary Materials 1.7 and 4; II-4, 5 years younger than the proband—Supplementary Materials 1.8 and 5; II-5, 14 years younger than the proband—Supplementary Materials 1.9 and 6), respectively (Fig. 1). Besides a mild light sensitivity in the father (I-1), there were no major relevant symptoms in the parents; the demonstrated slight cognitive impairment may have other explanations unrelated to XPA (Supplementary Material 1.2).


ESM 3(AVI 5946 kb)
ESM 4(AVI 8528 kb)
ESM 5(AVI 7504 kb)
ESM 6(AVI 4607 kb)


### Dermatological and dermatopathological assessments

Dermatological alterations generally include mild-to-moderate solar damage on sun-exposed areas with the presence of hyperpigmentation in the basal keratinocytes and solid and infiltrative basal cell carcinomas (Supplementary Material 1.10).

The results of the neurological and dermatological examinations enabled staging of all 5 siblings according to the Japanese Dermatological Association’s guideline (Table 1; [3]).

**Table 1:** The staging of XPA patients according to the classification of the severity of XP proposed by the Japanese Dermatological Association’s guidelines [3]

	Patient II-1	Patient II-2	Patient II-3	Patient II-4	Patient II-5
Cutaneous symptom (D) score					
Exaggerated sunburn	3	3	3	3	3
Freckle-like eruption	3	3	1	2	0
Skin cancer	3	2	0	0	0
Severity of cutaneous symptoms	D3	D3	D2	D2	D2
Extracutaneous symptom (N) score					
Hearing ability	1	N.A.	1	0	0
Movement	2	3	2	2	2
Intellectual functions	2	3	2	2	2
Swallowing and respiratory function	0	3	0	0	0
Severity of extracutaneous symptoms	N3	N3	N3	N2	N2
Classification of XP depending on the severity					
Stage	4	4	4	3	3

*XP*, xeroderma pigmentosum; *N.A.*, not available

### Ophthalmological assessment

Ophthalmological examinations demonstrated exophoria and weakness of convergence with gaze-dependent diplopia in patients II-1 and II-2. Pathological alterations of the vitreo-macular interface, i.e., thickened internal limiting membrane (ILM) with ruffled inner surface in the macular area of the retina, were diagnosed in patients II-2, II-3, and II-4. This sign can be considered as a gliotic proliferation of the inner surface of the ILM, although epiretinal membrane formation could not be detected.

### Diagnostic challenges and genomic studies

The major diagnostic challenges were that besides the severe neurological symptoms and signs, the dermatological alterations were not so prominent to be easily recognized by a neurologist.

Variants obtained by exome sequencing (for details, please see Supplementary Material 1.11) were filtered based on severity and frequency against public variant databases including dbSNP, ClinVar, ExAC, EVS, GnomAD, and an in-house clinical exome database of 300 unrelated Hungarian samples.

For the two new XPA variants (NM_000380.3:c.438_443delAGAATA; NP_000371.1:p.Gln146_Tyr148delinsHis and NM_000380.3:c.772_785delCGTAAGACTTGTAC; NP_000371.1:p.Arg258TyrfsTer5), ClinVar accessions VCV000523609.1 and VCV000523609.1 were assigned, respectively. The minor allele frequency (MAF) for *XPA* c.438_443delAGAATA variant is unknown since it is not listed in either the gnomAD (https://gnomad.broadinstitute.org/), ExAc (http://exac.broadinstitute.org/), or in the EVS (https://evs.gs.washington.edu/EVS/) databases. The MAF for *XPA* c.772_785delCGTAAGACTTGTAC variant is 0.0000814 (23/282538 allele) according to the gnomAD (https://gnomad.broadinstitute.org/) database, which is within the range for a pathogenic recessive allele. Based on the American College of Medical Genetics and Genomics variant interpretation guidelines [5], the first variant was classified as likely pathogenic, whereas the second as pathogenic. The conservation of the region of XPA protein affected by the in-frame mutation is demonstrated by Supplementary Material 1.12.

## Discussion

Regarding genotype-phenotype correlation, mutations affecting exons 2, 3 and introns 1, 3 in the *XPA* gene are almost always accompanied by severe characteristic clinical signs, whereas mutation sites approaching the C-terminal of the corresponding XPA protein may be considered as hypomorphic, i.e., they can be characterized by less severe neurological and especially, dermatological phenotypes [6, 7] (the clinical and demographic characteristics of all the published mutations in the *XPA* gene were collected and outlined in Supplementary Material 1.13). Indeed, cutaneous signs may be absent as well [8]. In addition to the mutation site, the clinical phenotype may be influenced by the age of the patient and, for dermatological and ophthalmological alterations, by sun exposure as well, even within the same family [9].

This genotype-phenotype correlation was confirmed by the current study as well, i.e., only mild-to-moderate dermatological and no prominent ophthalmological and audiological, but severe neurological signs evolved with age.

Despite the hypomorphic character of the reported variants, the likely pathogenic feature of the in-frame mutation in exon 4 (p.Gln146_Tyr148delinsHis) is supported by that it affects a conserved region of the protein (presented in Supplementary Material 1.12) including p.Glu147 and p.Tyr148, the deletion of which results in the pathologically reduced binding of XPA protein to replication protein A [10]. The second one in exon 6 (p.Arg258TyrfsTer5) may be considered pathogenic as it eventuates a premature stop codon. In addition to its frameshift character, the pathogenicity of this variant may be further supported by a later unrelated report of it in a patient with XPA (https://www.ncbi.nlm.nih.gov/clinvar/variation/523608/).

Furthermore, in addition to the presentation of a very detailed phenotypic description from a neurological point of view, the present study yields several novelties: (1) 5 out of 5 affected siblings within a single family has not been reported before, and this study presents the first cases of XPA in the Central-Eastern region of Europe. (2) Likely pathogenic novel in-frame and pathogenic novel frameshift mutations in trans position are presented. (3) A previously unreported scattered infiltration of CD8+ T lymphocytes was detected in the proband with XPA, without a manifest corresponding dermatological alteration. In light of the fact that in common neurodegenerative diseases with accumulation of proteins, CD8+ cytotoxic T cells are not considered as hallmark lesions; this finding is unexpected and may be part of an immunological activation following an insult from a currently uncharacterized agent or process. However, the significance of this observation could not be clarified in the present study and merits further observations on similar cases.

In conclusion, the presentation of this unusual neurological predominant phenotype of XPA may help clinicians recognize this rare disorder.

## Electronic supplementary material

ESM 1 (PDF 5237 kb)
