# Supplementary material for: Predominant neurological phenotype in a Hungarian family with two novel mutations in the XPA gene—case series
Source: Neurol Sci. 2019 Sep 2;41(1):125–9. doi: 10.1007/s10072-019-04044-6 (PMC6940312; doi:10.1007/s10072-019-04044-6)
Supplement: Supplementary file 6 — (PDF 5237 kb) [file 10072_2019_4044_MOESM1_ESM.pdf]

Neurological Sciences

Supplementary Material 1

Predominant neurological phenotype in a Hungarian family with two novel mutations in the *XPA* gene - case series

Authors: **Dénes Zádori[1], László Szpisjak[1], István Balázs Németh[2], Zita Reisz[3], Gabor G. Kovacs[4], Noémi Szépfalusi[1], Viola Luca Németh[1], Zoltán Maróti[5], Edit Tóth-Molnár[6], Judit Oláh[2], László Vécsei[1,7,8], Péter Klivényi[1], Tibor Kalmár[5]***

Affiliations:

[1]Department of Neurology, Faculty of Medicine, Albert Szent-Györgyi Clinical Center, University of Szeged, Szeged, Hungary

[2]Department of Dermatology and Allergology, Faculty of Medicine, Albert Szent-Györgyi Clinical Center, University of Szeged, Szeged, Hungary

[3]Department of Pathology, Faculty of Medicine, Albert Szent-Györgyi Clinical Center, University of Szeged, Szeged, Hungary

[4]Institute of Neurology, Medical University of Vienna, Vienna, Austria

[5]Genetic Diagnostic Laboratory, Department of Pediatrics and Pediatric Health Center, University of Szeged, Szeged, Hungary

[6]Department of Ophthalmology, Faculty of Medicine, Albert Szent-Györgyi Clinical Center, University of Szeged, Szeged, Hungary

[7]MTA-SZTE Neuroscience Research Group, University of Szeged, Szeged, Hungary

[8]Interdisciplinary Excellence Center, University of Szeged, Szeged, Hungary

***Corresponding author:**

Tibor Kalmár MSc, PhD

Genetic Diagnostic Laboratory, Department of Pediatrics and Pediatric Health Center, University of Szeged, Szeged, Hungary

H-6720 Szeged, Korányi fasor 14-15.

Phone: +36-62-341-711

E-mail: kalmar.tibor@med.u-szeged.hu

Supplementary Material 1.1

Legend for Supplementary Material 2. The video demonstrating the neurological assessment of patient II-2.

Supplementary Material 1.2

Cognitive characterization of patients with XPA.

	Father	Mother	Patient II-1	Patient II-2	Patient II-3	Patient II-4	Patient II-5
**Age at examination (years)**	62	60	42	39	36	34	25
**Years of education**	17	11	10	11	13	12	12
**Estimated premorbid IQ**							
**NART (max. 100%)**	87%	81%	82%	-	57%	63%	67%
**Degree of overall impairment**							
**MMSE (max. 30 p)**	30	28	24	19	26	27	28
**ACE (max. 100 p)**	89	84	63.5	35	64	70	87
**Severity of impairment**	-	+	++	+++	++	++	-
**Verbal memory**							
**ACE anterograde memory (max. 7 p)**	5	0	0	0	1	3	7
**RBMT story delayed recall (max. 21 p)**	5	6.5	0.5	-	3	0	4
**Severity of impairment**	+	++	+++	+++	++	++	+
**Non-verbal memory**							
**RBMT face recognition delayed recall (max. 10 p)**	10	9	10	5	8	8	9

**RBMT picture recognition delayed recall (max. 20 p)**	20	20	19	8	20	20	20
**Severity of impairment**	-	-	-	+++	-	-	-
**Simple working memory**							
**DST**	8	6	5	-	5	5	6
**RBMT story immediate recall (max. 21 p)**	11	7.5	2.5	-	4.5	0	6
**CBTT**	4	4	4	-	4	3	3
**Severity of impairment**	-	-	+	N.A.	+	+	+
**Complex working memory**							
**LST**	3	2.66	1.66	-	1.66	1.66	2
**BDST**	5	4	4	-	3	3	4
**Severity of impairment**	-	+	++	N.A.	++	++	+
**Language**							
**ACE language (max. 28 p)**	27	28	28	26	22	26	28
**Severity of impairment**	+	-	-	+	++	+	-
**Attention**							
**Pieron test N (max. 400 p)**	200	237	123	180	200	60	130
**Pieron test T% (max. 100%)**	98	96	84	51	81	78	96
**Severity of impairment**	-	-	+	+++	+	++	-
**Executive functions**							
**Letter fluency**	6.33	11	7.66	-	6.66	8.33	8.66

**Semantic fluency**	15	19.5	12	-	8.5	7.5	14.5
**Episodic fluency**	18	19	16	-	9	9	15
**Verb fluency**	19	16	14	-	4	7	14
**TMT Part B (sec)**	192	75	520	-	321	690	143
**TMT Part B error**	3	0	7	-	4	10	1
**Severity of impairment**	++	-	+++	N.A.	++	+++	++
**Visuospatial skills**							
**BVMT-R (max. 12 p)**	12	12	10	6	12	12	12
**ACE figure drawing (max. 2 p)**	2	2	2	0	2	1	1
**Severity of impairment**	-	-	+	+++	-	+	+
**Processing speed**							
**TMT Part A (sec)**	63	43	116	-	88	105	43
**TMT Part A error**	0	0	0	-	0	0	0
**Severity of impairment**	++	+	++	N.A.	+++	+++	+
**Mood**							
**BDI (max. 63 p)**	8	7	29	-	8	7	6
**Severity of impairment**	-	-	+++	N.A.	-	-	-
**Anxiety**							
**STAI-S**	32	38	47	-	30	37	41
**STAI-T**	37	46	49	-	36	45	35
**Severity of impairment**	-	-	-	N.A.	-	-	-

The demonstrated cognitive dysfunction is caused by the impairment of two functional neuroanatomical networks, the hippocampus-dependent and that related to the prefronto-cerebellar system, with similar degrees of impairment. The mother has an overall slight (ACE: 84 points) cognitive disability which is seemingly attributed to her poor performance in anterograde verbal memory test. This alteration may be the consequence of regular benzodiazepine use. Despite the lack of severe depression or anxiety, the patient takes this medication to keep with the stressful events related to the care of her disabled children. Regarding the father, the impairment in overall cognitive functions is not clinically significant (ACE: 89 points, MMSE: 30 points), but the executive functions and processing speed are seemingly decreased. The reason behind that may be the presence of severe insomnia accompanied with occasional daytime somnolence. Nevertheless, both parents should be controlled for further progression.

ACE: Addenbrooke's Cognitive Examination, BDI: Beck Depression Inventory, BDST: Backward Digit Span Task, BVMT-R: Brief Visuospatial Memory Test-Revised, CBTT: Corsi Block-Tapping Test, DST: Digit Span Task, LST: Listening Span Task, MMSE: Mini-Mental State Examination, N.A.: not available, NART: National Adult Reading Test, RBMT: Rivermead Behavioural Memory Test, STAI: The State-Trait Anxiety Inventory, TMT: Trail Making Test

Supplementary Material 1.3

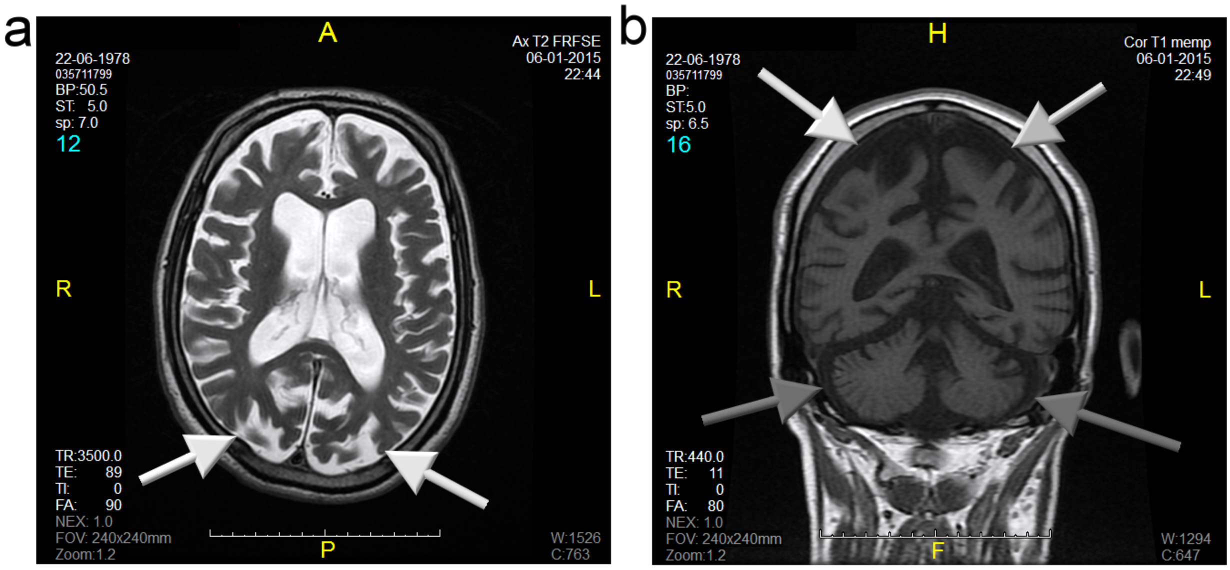

Brain magnetic resonance imaging of the proband with XPA. T2-weighted axial images (1.5 Tesla) (**a**) and T1-weighted coronal images (1.5 Tesla) (**b**) demonstrate generalized brain atrophy with slight predominance at the parieto-occipital region (indicated with white arrows) and in the cerebellum (indicated with gray arrows) in patient II-2.

Supplementary Material 1.4

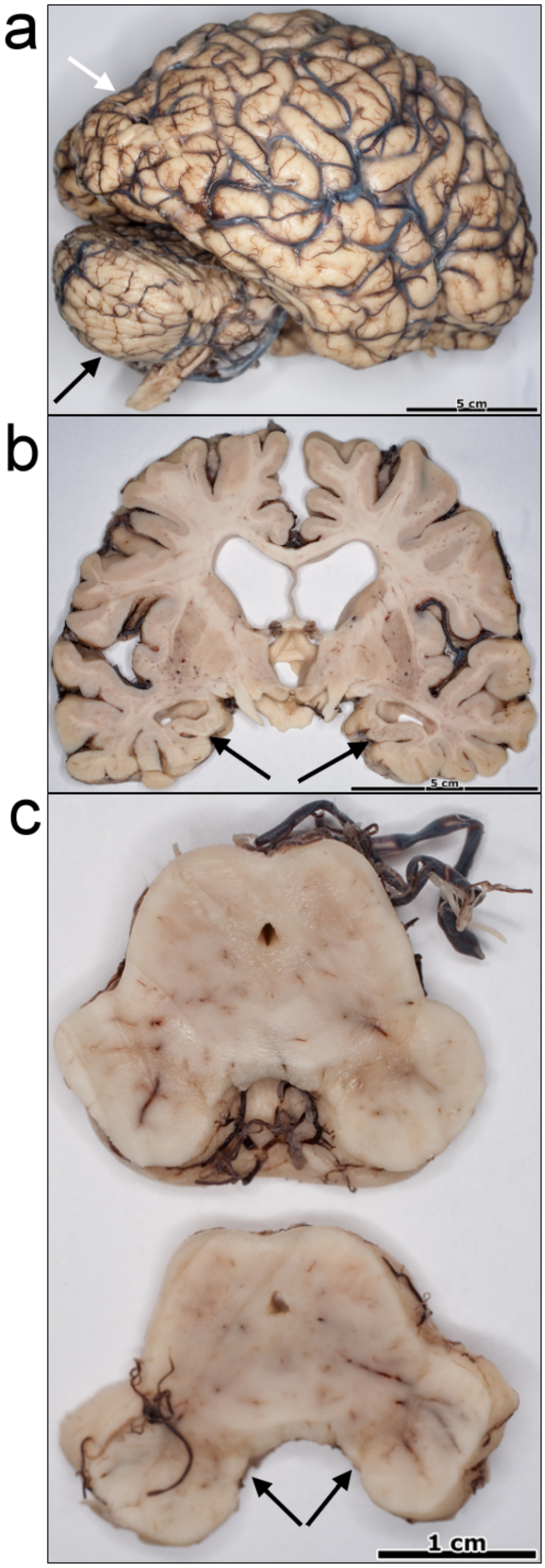

Macroscopic neuropathological alterations of the proband with XPA. The total brain weight was 815 grams, the cerebellum-brainstem weight was 115 grams. The external examination of the hemispheres, especially that of the parieto-occipital region (**a**; white arrow), and the cerebellum-brainstem (**a**; black arrow) revealed marked atrophy. On the coronal sections a prominent thinning of the cortical ribbon and the cerebellar folia could be seen and the volume of the white matter was also decreased. The hippocampus was markedly atrophic on both sides (**b**; black arrows). The heads of the caudate nuclei were symmetrically flattened. The thalamus and lentiform nuclei showed mild atrophy. A remarkable ventricular dilatation and spacing in the Sylvian fissure could be seen. The cross-sections of the brainstem revealed an almost complete discoloration of the substantia nigra (**c**; black arrows). The anterior and posterior horn of the spinal cord and the spinal nerves were proportionately atrophic. In addition to the marked atrophy behind considerably decreased brain weight, the craniometric measurements may raise the possibility of slight microcephaly in patients II-1 and II-2, but no microcephaly could be demonstrated in other family members.

Supplementary Material 1.5

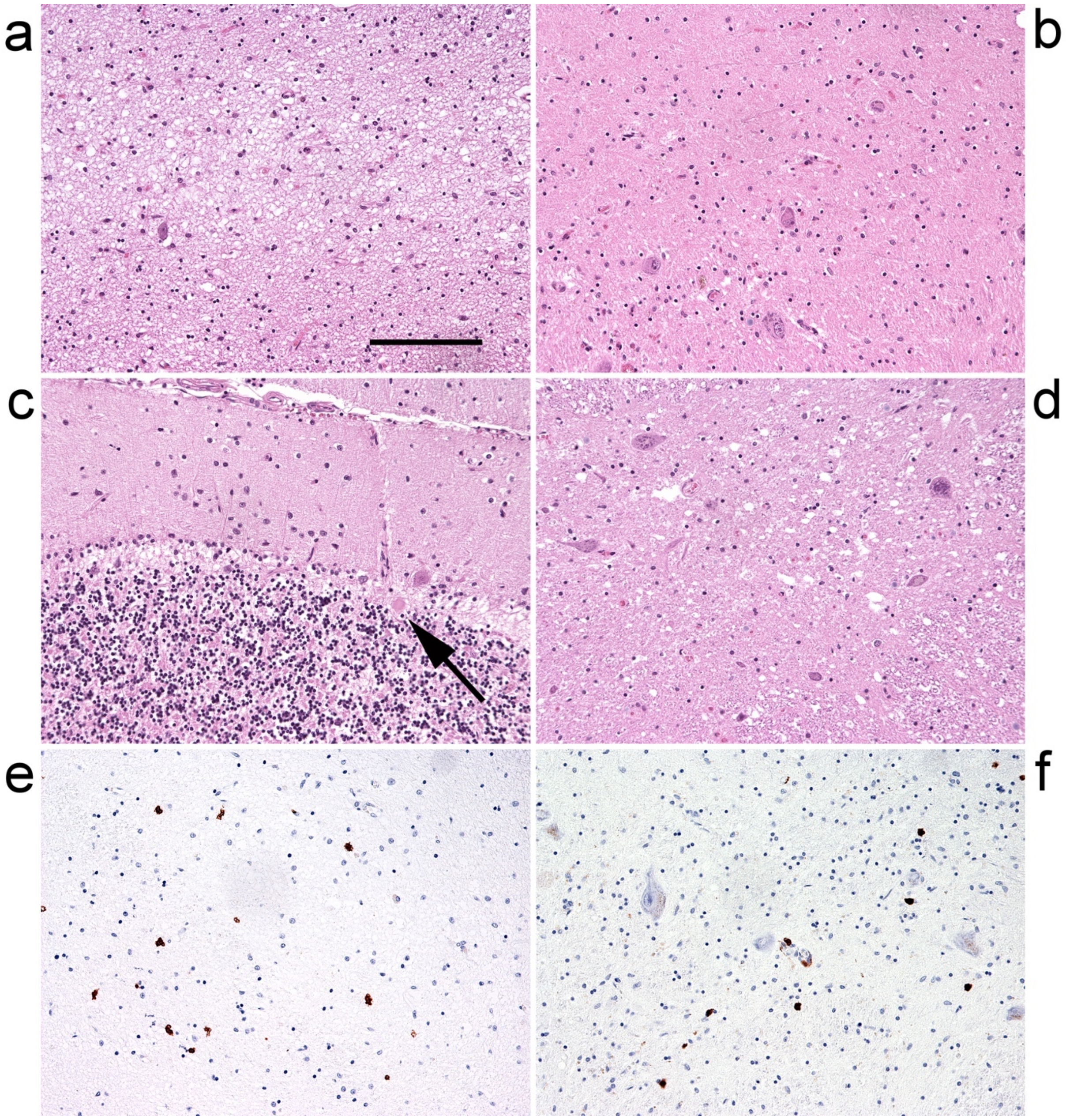

Microscopic *post mortem* neuropathological alterations of the proband with XPA. Formalin-fixed (*post mortem* delay of 600 hours), paraffin-embedded tissue blocks (2.5 x 2.0 cm) were applied. The presence of astrogliosis/microgliosis/neuronal loss, and degree of various protein depositions were semi-quantitatively (none, mild, moderate, severe) evaluated in the following anatomical regions: frontal, temporal, occipital and premotor cortex, basal ganglia and thalamus, amygdala, hippocampus and brainstem levels (mesencephalon, pons, medulla oblongata). Histological examination revealed mild loss of neurons with edema-related spongy loosening of the neuropil in the superficial layers of cortical areas. There was a lack of ballooned neurons or eosinophilic inclusion bodies. The basal ganglia, thalamus and amygdala were also relatively well preserved. In the hippocampus we observed prominent loss of neurons and reactive astrogliosis in the CA1

(**a**; H&E staining) and CA4 subregions. This was asymmetrical and involved mostly the left side. Further mild to moderate neuronal loss was seen in the substantia nigra (**b**; H&E staining) and severe loss was observed in the cerebellum (**c**; H&E staining, arrow indicates a torpedo), where the Purkinje cell layer showed significant depletion with Bergmann-gliosis and formation of axonal torpedos. Other brainstem nuclei did not show relevant neuronal loss, except for moderate gliosis in the inferior olives. The anterior horn motor neurons were moderately depleted (**d**; H&E staining). There was a lack of vascular lesions in the examined areas. Microglial activation was seen in areas with neuronal loss and additionally scattered CD8 positive cytotoxic T cells, but no CD20 positive B cells, were also observed in the hippocampus (**e**), substantia nigra (**f**), cerebellar white matter and dentate nucleus. In further anatomical regions mild CD8 positive cell infiltrations were seen around vessels in the white matter. Immunostaining for α-synuclein, amyloid-beta, TDP-43, phospho-TDP-43, ubiquitin and p62 did not reveal pathological protein deposits in any of these regions. In the immunostaining for phospho-tau only a single neuron was seen in the entorhinal cortex on the side where sclerosis was observed.
The bar in (**a**) represents 50 micrometer for all images.

Method for immunohistochemistry: In addition to Hematoxylin and Eosin and Luxol Fast Red, the following monoclonal antibodies were used for immunohistochemistry: anti-tau AT8 (pS202/pT205, 1:200, Pierce Biotechnology, Rockford, IL, USA), anti-phospho-TDP-43 (pS409/410, 1:2,000, Cosmo Bio, Tokyo, Japan), anti-α-synuclein (1:2,000, clone 5G4, Roboscreen, Leipzig, Germany), anti-Aβ (1:50, clone 6F/3D, Dako, Glostrup, Denmark), anti-p62 (1:1,000, BD Transduction, Lexington KY, USA), anti-ubiquitin (1:50,000, Millipore, Temecula, CA, USA), TDP-43 (1:2,000, Abnova Corp., Taipei City, Taiwan), anti-CD8 (1:100, DakoCytomation, Glostrup, Denmark), and anti-HLA-DR (clone CR3/43, 1:100, DakoCytomation, Glostrup, Denmark). The DAKO EnVision© detection kit, peroxidase/DAB, rabbit/mouse (Dako, Glostrup, Denmark) was used for visualization of antibody reactions.

Supplementary Material 1.6

Detailed description of the neurological and related alterations of patient II-1 and legend for Supplementary Material 3. Besides the presence of an exaggerated sunburn reaction from her infancy, the symptoms of patient II-1 became manifest at the beginning of her 30s. She presented memory problems, as well as speech and gait disturbances.
Regarding the neurological and neuropsychological examination of patient II-1, the following alterations were observed: disturbed eye movements (exophoria, saccadic eye movements with

restricted gaze in the upward direction), dysarthria, hypo-/areflexia, bilateral palmomental sign, movement disorder with dominating ataxia (bilateral dysmetria, cerebellar predominant mixed limb ataxia more pronounced in the legs, truncal ataxia, severe postural instability, broad-based, ataxic gait, and mild postural tremor), decreased sense of vibration, moderate cognitive dysfunction and severe depression (Supplementary Material 1.2; The video demonstrating the neurological assessment of patient II-1 is uploaded as Supplementary Material 3).
The brain MRI revealed generalized atrophy with slight predominance regarding the parieto-occipital and cerebellar structures.
Audiological screening demonstrated only slight sensorineural hearing impairment at low frequencies.

Supplementary Material 1.7

Detailed description of the neurological and related alterations of patient II-3 and legend for Supplementary Material 4. The neurological symptoms of patient II-3 became manifest at the beginning of his 30s. Upon examination he presented disturbed eye movements (saccadic eye movements with restricted gaze in the upward direction), dysarthria, hypo-/areflexia, slight muscle hypotonia, bilateral palmomental sign, movement disorder with dominating ataxia (mainly left-sided bilateral dysmetria, cerebellar predominant mixed limb ataxia more pronounced in the legs, truncal ataxia, severe postural instability, broad-based, ataxic gait, mild postural tremor, occasional spontaneous distal myoclonic jerks which can be exacerbated by sensory stimuli thereby presumed to be cortical origin) and moderate cognitive dysfunction (Supplementary Material 1.2; The video demonstrating the neurological assessment of patient II-3 is uploaded as Supplementary Material 4). Only a slightly exaggerated sunburn reaction was present.
Audiological screening demonstrated slight hearing impairment at low frequencies on the left side and at high frequencies on the right side.

Supplementary Material 1.8

Detailed description of the neurological and related alterations of patient II-4 and legend for Supplementary Material 5. In addition to the presence of pronounced light sensitivity, complicated scar healing and disturbed vision, the neurological symptoms of patient II-4 became manifest at approximately in the middle of his 20s. Upon examination disturbed eye movements (saccadic eye movements with restricted gaze in the upward direction, gaze-evoked nystagmus), dysarthria, hypo-/areflexia, bilateral palmomental sign, movement disorder with dominating ataxia (bilateral dysmetria, cerebellar predominant mixed limb ataxia more pronounced in the legs, truncal ataxia, severe postural instability, broad-based, ataxic gait, mild postural tremor,

slight left-sided bradykinesia), parietal lobe signs (left-right disorientation, dressing apraxia), decreased sense of vibration and moderate cognitive dysfunction were detected (Supplementary Material 1.2; The video demonstrating the neurological assessment of patient II-4 is uploaded as Supplementary Material 5).

The brain MRI revealed atrophy predominantly affecting the parieto-occipital and cerebellar structures.

Electroneurography demonstrated mixed sensorimotor lower limb predominant polyneuropathy.

Audiological screening did not show any hearing abnormality.

Supplementary Material 1.9

Detailed description of the neurological and related alterations of patient II-5 and legend for Supplementary Material 6. In addition to pronounced light sensitivity and slightly disturbed speech, patient II-5 does not have any major complaint. The neurological examination of patient II-5 identified mainly minor alterations: saccadic eye movements, slight dysarthria, hyporeflexia, palmomental sign on the left side, cerebellar predominant mixed limb ataxia more pronounced in the legs, slightly broad-based, ataxic gait, and only mild-to-moderate cognitive impairment (Supplementary Material 1.2; The video demonstrating the neurological assessment of patient II-5 is uploaded as Supplementary Material 6).

The brain MRI demonstrated only slight atrophy mainly affecting the parieto-occipital region and the cerebellum.

Audiological screening did not show any hearing abnormality.

Supplementary Material 1.10

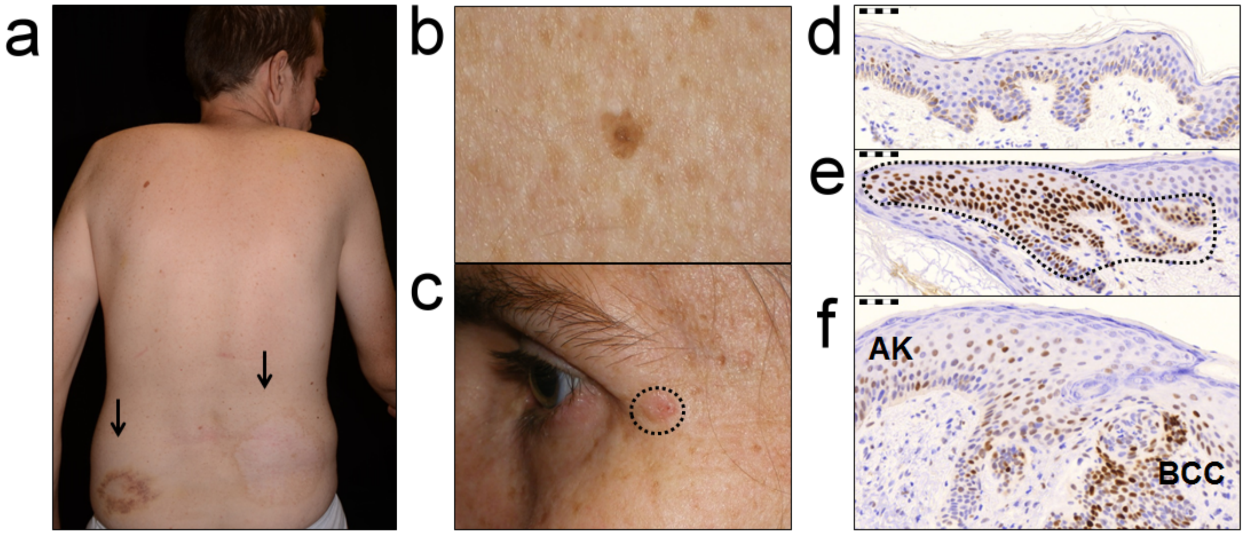

Representative clinical and histopathological pictures of dermatological alterations of the XPA family members. Among the family members, solar lentigos and lentiginous naevi were more numerous in patients II-1 and II-2, and scattered foci of basal cell carcinoma appeared and were excised from the nasal and periorbital areas. Patient II-2 exhibited solar lentigos on the upper back and shoulder areas, note the foci of healing suffusions resulting from previous traumatisation (**a** - arrows). Patient II-1 has multiple solar lentigos affecting diffuse facial, as well as trunk and arm regions; dotted line marks a focus of nodular basal cell carcinoma (**b**, **c**). Histopathologically, solar affected back skin shows basal hyperpigmentation in the keratinocytes with some elongation of the rete ridges (**d**), and with foci of p53 mutated clones (**e** – dot mark). In addition of actinic keratosis (AK), basal cell carcinoma (BCC) nests from facials tumours have p53 protein accumulation as well (**f**, IHC, brown colorimetric reaction; OM 112x). In the younger brothers, only lentigos were noted on the sun-exposed skin. Written informed consent was obtained from the participants of the study for the publication of photo materials with identifying information. The bars in (**a**), (**b**) and (**c**) represent 50 micrometer.

Method for immunohistochemistry: Following punch biopsy of the sun-exposed skin, p53 immunohistochemistry (clone PA0057 ready to use dilution with heat induced epitope retrieval at pH = 9 for 30 minutes; Leica Biosystems, UK) was performed.

Supplementary Material 1.11

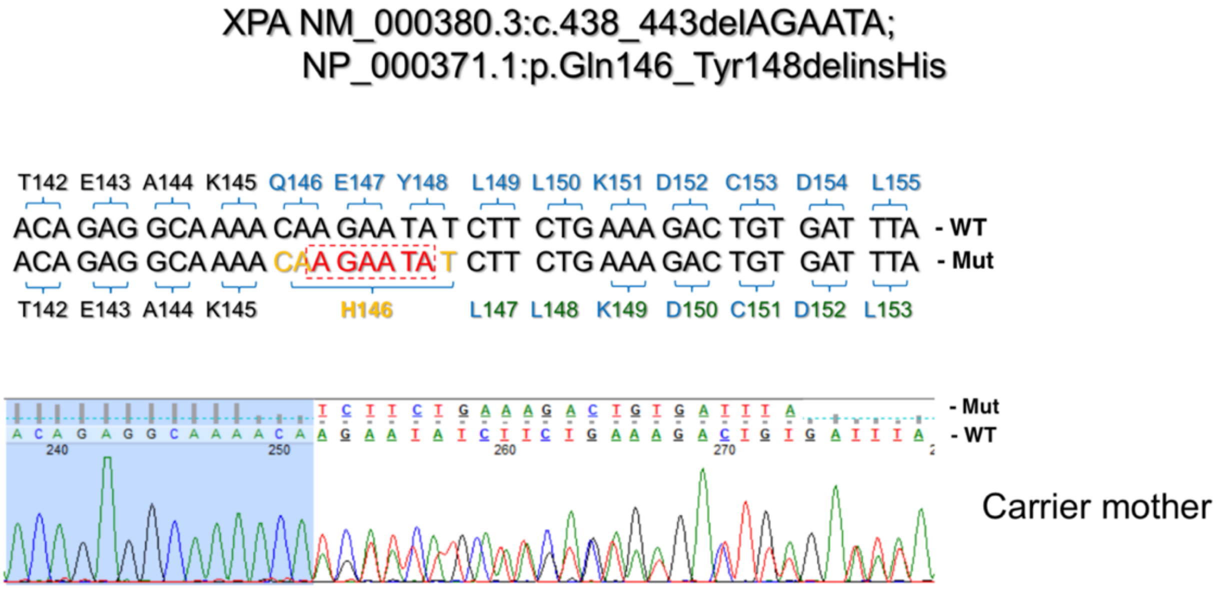

Part of XPA Exon 4

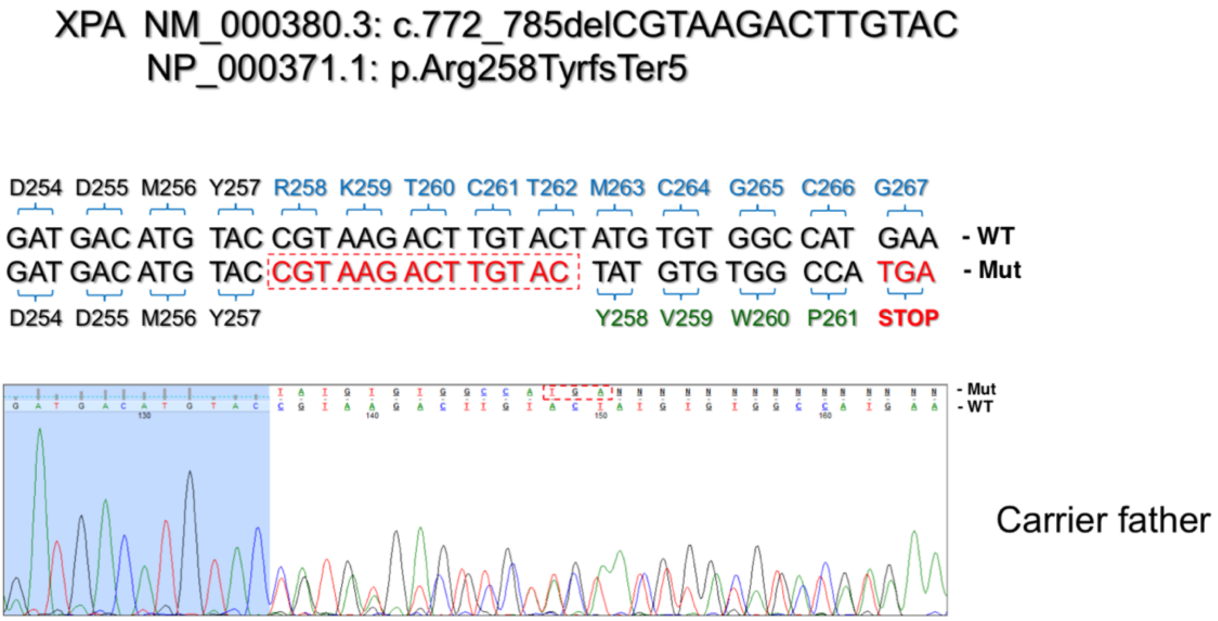

Part of XPA Exon 6

Sanger sequencing of the identified XPA mutations in the carrier parents and the detailed description of genomic studies. In light of dominating cerebellar and cognitive dysfunction and the pseudo-dominant pattern of inheritance the main causes of dominantly inherited ataxia (*ATXN1*, *ATXN2*, *ATXN3* and *TBP* genes) and cognitive dysfunction (*FMR1*, *PSEN1*, *PSEN2* and *APP* genes) were assessed first, finding no relevant alteration. Therefore, targeted gene analysis by clinical exome sequencing was performed to find the pathogenic alteration behind the severe clinical phenotype. After obtaining written, informed consent, genomic DNA was extracted from peripheral blood leukocytes by standard protocol. For clinical exome sequencing, a total of 60 ng

of genomic DNA was used for library preparation and sequenced with TruSight One clinical exome kit (Illumina) on Illumina MiSeq platform. The clinical exome kit covers the coding region of 4813 clinically relevant, disease-associated genes. The 150-bp paired reads were aligned to the GRCh37.75 human reference genome by Burrows Wheel Aligner (BWA v0.7.9a) software. The variants were called by Genome Analysis Toolkit Haplotype Caller (GATK v3.5) best practice and annotated by SnpEff and VariantStudio softwares.
Parents of the index patient were tested for phase establishment. We could confirm that the 2 pathogenic variants identified in the index patient were in trans phase since the mother was heterozygous for the variant in exon 4, whereas the father was heterozygous for the variant in exon 6 of the XPA gene. The likely pathogenic feature of the in-frame mutation in exon 4 (p.Gln146_Tyr148delinsHis) is supported by that it affects a conserved region of the protein (presented in Supplementary Material 1.12) including p.Glu147 and p.Tyr148, the deletion of which results in the pathologically reduced binding of XPA to replication protein A. The second one in exon 6 (p.Arg258TyrfsTer5) maybe considered pathogenic as it eventuates a premature stop codon. The alterations found by clinical exome sequencing were also confirmed by Sanger sequencing in cases of all available family members (the variants of carrier parents are presented above).

Supplementary Material 1.12

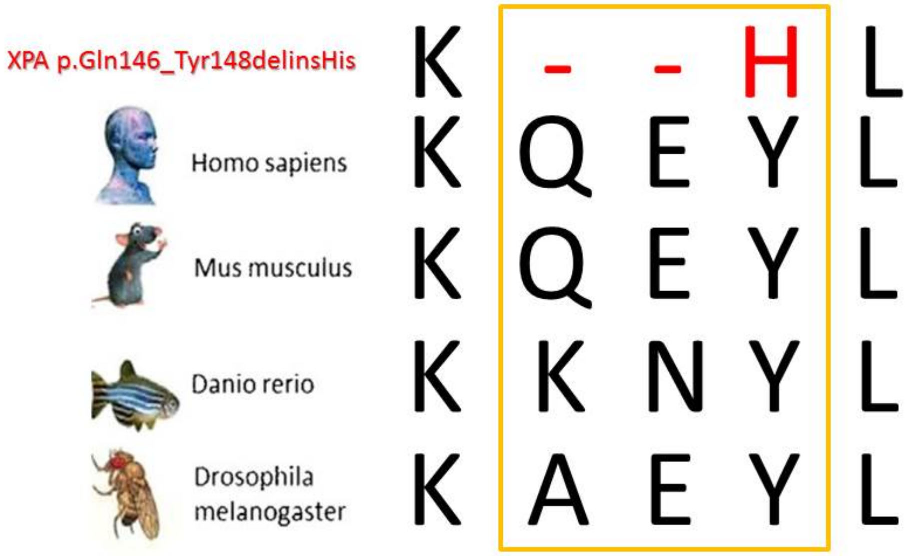

The figure demonstrates that the in-frame mutation is located in a conserved region of the XPA protein.

Supplementary Material 1.13

The demographic and clinical characteristics of patients with mutations in the *XPA* gene.

Mutation (DNA)	Mutation (Protein)	Exon/ Intron	Case number	Nation	Gender (M/F)	Age of onset (years)	Skin symptoms	Cancer	Mental retardation	Cerebellar symptoms	Pyramidal signs	Hearing impairment	Ocular signs	References
c.172+2T>G + c.390-1G>C	Int. 1 + Int. 3	Int. 1 Int. 3	1	Japanese	1/0	<1	+++	-	-	-	-	NA	NA	1
c.253C>T	p.Gln85Ter	2	1	Bangladeshi	0/1	5#	++	-	++	++	++	NA	+	2
c.266_267dup AA	p.Val90fs	2	1	United Kingdom	1/0	<1	++	-	++	++	++	NA	+	2
c.268_269insA A	p.Val90KLysfs*14	2	1	Moroccan	1/0	<1	+++	P	+++	NA	NA	NA	NA	3
c.283G>A	p.Gly95Arg	2	1	Finnish	ND	<1	+++	P	P	P	-	-	+	4
c.283G>A + c.631C>T	p.Gly95Arg + p.Arg211Ter	2, 5	1	Chinese	1/0	<1	P	-	P	P	-	P	P	5
c.314G>A	p.Cys105Tyr	3	2	Somali	1/1	8, 12#	+/++	-	++	++	++	NA	+	2
c.323G>T + c.349_353delC TTAT	p.Cys108Phe + p.Lys117Glufs*4	3	1	Caucasian	NA	NA	NA	NA	NA	NA	NA	NA	NA	6
c.331G>T	p.Glu111Ter	3	4	3 Tunisian, 1 Egyptian	3/1	<1	+++	P	+++	-: 1<br>++: 2<br>+++: 1	P	NA	P: 2	7, 8
c.331G>T + c.389+1G>C	p.Glu111Ter + Int. 3	3, Int. 3	1	Chinese	1/0	<1	P	-	P	P	-	P	P	5
c.335_338delT TATinsCATA AGAAA	p.Phe112Serfs*2	3	2	Indian	0/2	<1	P	-	P	P	P	P: 1<br>-: 1	P	9
c.348T>A	p.Tyr116Ter	3	1	Japanese	0/1	<1	+++	P	+++	P	P	NA	NA	10
c.348T>A + c.390-1G>C	p.Tyr116Ter + Int. 3	3, Int. 3	4	Japanese	M: 2<br>NA: 2	early childhood	+: 1<br>+++: 2<br>P: 1	-: 1<br>P: 1<br>NA: 2	-: 1<br>+++: 3	-: 1<br>++: 1<br>+++: 2	-: 1<br>+++: 2<br>NA: 1	P: 1<br>NA: 3	+: 1<br>NA: 3	11, 12, 13

c.349_353delC TTAT	p.Leu117Glufs*4	3	3	2 Indian, 1 Iranian	1/2	<1	++: 1<br>P: 2	-: 2<br>NA: 1	P	P: 2<br>NA: 1	-: 2<br>NA: 1	-	++: 1<br>P: 2	9, 14
c.374delC	p.Pro125Ilefs*15	3	2	1 Caucasian,<br>1 Egyptian	F: 1<br>NA: 1	<1, NA	+: 1<br>NA: 1	-: 1<br>NA: 1	+: 1<br>NA: 1	-: 1<br>NA: 1	-: 1<br>NA: 1	NA	+: 1<br>NA: 1	6, 8
c.378T>G	p.Cys126Trp	3	1	Indian	1/0	<1	P	-	P	P	P	-	P	9
c.389G>A	p.Arg130Lys	3	2	Black (breed)	NA	NA	++: 1<br>NA: 1	-: 1<br>NA: 1	NA	NA	NA	NA	P: 1<br>NA: 1	15
c.389+1G>T +<br>c.631C>T	Int. 3 +<br>p.Arg211Ter	5, Int. 3	1	Chinese	1/0	<1	P	P	P	P	-	P	-	5
c.390-1G>C	Int. 3	Int. 3	60	57 Japanese,<br>3 Chinese	28/16<br>NA: 16	<1: 42<br>3: 1<br>NA: 17	++: 18<br>+++: 15<br>P: 10<br>NA: 17	P: 15<br>-: 23<br>NA: 22	+: 4<br>++: 10<br>+++: 11<br>-: 12<br>P: 3<br>NA: 20	+: 4<br>++: 12<br>+++: 7<br>-: 12<br>P: 3<br>NA: 22	+: 4<br>++: 6<br>+++: 6<br>-: 13<br>NA: 31	P: 23<br>-: 15<br>NA: 22	+: 1<br>++: 15<br>+++: 10<br>P: 2<br>NA: 32	5, 11, 12, 13, 16, 17, 18, 19, 20, 21, 22
c.390-1G>T +<br>c.555G>C	Int. 3 +<br>p.Gln185His	4, Int. 3	1	Caucasian	NA	NA	NA	NA	NA	NA	NA	NA	NA	23
c.390-1G>C +<br>c.619C>T	Int. 3 +<br>p.Arg207Ter	5, Int. 3	1	Japanese	1/0	<1	+	-	+	+	+	-	+	11
c.390-1G>C +<br>c.622C>T	Int. 3 +<br>p.Gln208Ter	5, Int. 3	1	Japanese	1/0	<1	+++	-	+++	+++	-	P	P	24
c.390-1G>C +<br>c.682C>T	Int. 3 +<br>p.Arg228Ter	6, Int. 3	12	Japanese	2/7<br>NA: 3	<1: 8<br>4:1<br>NA: 3	+: 6<br>++: 4<br>P: 2	P: 4<br>-: 8	+: 6<br>++: 2<br>+++: 3<br>-: 1	+: 6<br>++:2<br>-: 4	+: 6<br>-: 1<br>NA: 5	P: 4<br>-: 4<br>NA: 4	+: 2<br>NA: 10	11, 12, 13, 17, 25, 26, 27
c.390-1G>C +<br>c.690insT	Int. 3 +<br>p.231Lysfs*15	6, Int. 3	1	Japanese	1/0	NA	+	-	+	+	-	NA	NA	28
c.390-1C>T +<br>c.779insTT +<br>c.780insTT	Int. 3 +<br>p.Thr260Ilefs*8	6, Int. 3	3	Japanese	1/2	NA	+	-	+	+	-	NA	NA	28
c.390-1G>C +<br>?	Int. 3 + ?	Int. 3 +<br>?	3	Japanese	1/2	<1	P	P: 1<br>-: 2	++: 1<br>+++: 1<br>-: 1	++: 2<br>+++: 1	NA	+	NA	13

c.428_429delAG	p.Glu143Glyfs*11	4	2	Indian	NA	<1	P	-	P	P	P: 1<br>-: 1	P	P	9
**c.438_443delAGAATA + c.772_785delCGTAAGACTTGTAC**	**p.Gln146_Tyr148del_insHis + p.Arg258Tyrfs*5**	**4, 6**	**5**	**Hungarian**	**4/1**	**13-14: 1<br>~25: 2<br>~30: 2**	**+: 3<br>++: 2**	**P: 2<br>-: 3**	**++: 3<br>+++: 1<br>-: 1**	**+++: 4<br>+: 1**	**+: 1<br>-: 4**	**+: 2<br>-: 2<br>NA: 1**	**-: 5**	
c.451A>T	p.Lys151Ter	4	1	Chinese	0/1	<1	+	-	P	NA	NA	-	-	29
c.467_486del20nt + c.665insA	p.Lys157Ilefs*13 + p.Val223Serfs*22	4, 5	2	NA	0/2	+	NA	-	-	-	-	-	-	30
c.482delC + c.538_539delCT	p.Pro161fs*6 + p.Tyr181Leufs*11	4	1	Chinese	1/0	1.5	P	-	P	P	-	P	P	5
c.529G>A	p.Asp177Asn	4	1	Japanese	0/1	62#	++	P	-	-	-	-	-	31
c.553C>T	p.Gln185Ter	4	2	Egyptian	0/2	<1	+++	P: 1<br>-: 1	-	-	P: 1<br>-:1	NA	++: 1<br>+++: 1	8
c.555G>C	p.Gln185His	4	1	Caucasian	NA	NA	+	NA	+	+	+	P	NA	15
c.555G>C + c.631C>T	p.Gln185His + p.Arg211Ter	4, 5	1	Caucasian	NA	NA	NA	NA	NA	NA	NA	NA	NA	15
c.555G>C + c.731A>G	p.Arg185His + p.His244Arg	4, 6	1	Caucasian	NA	NA	+	NA	+	+	+	P	NA	15
c.555+8A>G	Int. 4	Int. 4	13	9 Pakistani, 3 Indian, 1 Afghan	7/6	3: 1<br>4-46#: 12	+: 9<br>++: 2<br>-: 2	P: 3<br>-: 10	-	-	-	-	P: 1<br>-: 12	2, 32
c.609-2A>G	? ##	? ##	1	Northern Africa	NA	NA	NA	NA	NA	NA	NA	NA	NA	33

c.619C>T	p.Arg207Ter	5	4	1 Tunisian, 2 Brazilian, 1 Palestinian	1/2 NA: 1	<1: 3 NA: 1	+++	P: 2 -: 2	+: 2 +++: 2	+++	+++: 1 P: 1 -: 2	P: 1 NA: 3	+: 1 NA: 3	7, 12, 34, 35
c.619C>T + c.622C>T	p.Arg207Ter + p.Gln208Ter	5	2	Chinese	2/0	<1	+++	-	P	NA	NA	-	-	29
c.631C>T	p.Arg211Ter	5	3	Chinese	1/2	<1	+++: 1 P:2	P: 1 -: 2	P	P	-:2 NA: 1	P: 2 -: 1	P:2 NA: 1	5, 36
c.631C>T + c.682C>T	p.Arg211Ter + p.Arg228Ter	5, 6	1	Chinese	1/0	<1	P	-	P	P	-	P	P	5
c.640dupA	p.Met214fs	5	1	Bangladeshi	0/1	1[#]	+	-	++	++	++	NA	+	2
c.647_648delAG	p.Lys217Glufs*3	5	2	Chinese	2/0	<1	+: 1 +++: 1	P: 1 -: 1	P: 1 -: 1	P: 1 -: 1	P: 1 -: 1	P: 1 -: 1	P: 1 -: 1	18
c.654delA	p.Lys218Asnfs*5	5	5	Pakistani	4/1	<1	+++: 3 NA: 2	P: 2 -: 1 NA: 2	-: 3 NA: 2	-: 3 NA: 2	-: 3 NA: 2	-: 3 NA: 2	-: 3 NA: 2	37
c.682C>T	p.Arg228Ter	6	46	17 Tunisian, 10 Moroccan, 7 Northern Africa, 6 Finnish, 2 Pakistani, 2 Japanese, 2 Algerian	18/8 NA: 20	<1: 20 <3: 6 NA: 20	+: 15 ++: 11 +++: 8 -: 3 P: 2 NA: 7	P: 17 -: 22 NA: 7	+: 5 ++: 1 +++: 2 -: 6 P: 17 NA: 15	+: 1 ++: 1 +++: 1 -: 14 P: 10 NA: 19	+: 1 ++: 1 +++: 1 -: 14 P: 10 NA: 19	P:11 -: 11 NA: 24	+: 4 ++: 3 -: 4 P: 10 NA: 25	2, 3, 4, 12, 17, 33, 38, 39, 40, 41, 42
c.682C>T + c.722insG	p.Arg228Ter + p.Val241Glyfs*4	6	3	Tunisian	1/2	25[#], 47, 56	-	-	+	P: 2 -: 1	P: 2 -: 1	P: 1 -: 1 NA: 1	NA: 3	42
c.682C>T + ?	p.Arg228Ter + ?	6	2	Japanese	0/2	<1	P	-	++: 1 -: 1	++	NA	-	NA	13

#: Age at diagnosis.

##: Intronic mutation was proposed, but the given mutation site is exonic (Exon 5).

?: Unknown mutation.

+: mild; ++: moderate; +++: severe; -: absent; NA: not available; P: present, but not detailed, Int: Intron.

Methods

PubMed (MEDLINE) was used as search engine for articles. The key terms for searching were: xeroderma AND pigmentosum AND group A. A total of 3056 articles were screened for mutational spectrum of the *XPA* gene. Out of these papers, 42 contained relevant phenotypic information in connection to the reported genetic alterations. Accordingly, in addition to genetic data, the number of relevant cases, their nationality, gender, the age of onset, the severity of dermatological, ophthalmological, auditory and neurological symptoms and the presence or absence of skin malignancy are reported here.

References

1. Tanioka M, Budiyant A, Ueda T, Nagano T, Ichihashi M, Miyachi Y, et al (2005) A novel XPA gene mutation and its functional analysis in Japanese patient with xeroderma pigmentosum group A. J Invest Dermatol 125:244-6. https://doi.org/10.1111/j.0022-202X.2005.23783.x

2. Sethi M, Haque S, Fawcett H, Wing JF, Chandler N, Mohammed S et al (2016) A Distinct Genotype of XP Complementation Group A: Surprisingly Mild Phenotype Highly Prevalent in Northern India/Pakistan/Afghanistan. J Invest Dermatol 136:869-72. https://doi.org/10.1016/j.jid.2015.12.031

3. Lehmann J, Schubert S, Schäfer A, Laspe P, Haenssle HA, Ohlenbusch A, et al (2015) A novel mutation in the XPA gene results in two truncated protein variants and leads to a severe XP/neurological symptoms phenotype. J Eur Acad Dermatol Venereol 29:2479-82. https://doi.org/10.1111/jdv.12841

4. Anttinen A, Koulu L, Nikoskelainen E, Portin R, Kurki T, Erkinjuntti M, et al (2008) Neurological symptoms and natural course of xeroderma pigmentosum. Brain 131:1979-1989. https://doi.org/10.1093/brain/awn126

5. Zhou EY, Wang H, Lin Z, Xu G, Ma Z, Zhao J, et al (2017) Clinical and molecular epidemiological study of xeroderma pigmentosum in China: A case series of 19 patients. J Dermatol 44:71-75. https://doi.org/10.1111/1346-8138.13576

6. Satokata I, Tanaka K, Okada Y (1992) Molecular basis of group A xeroderma pigmentosum: a missense mutation and two deletions located in a zinc finger consensus sequence of the XPAC gene. Hum Genet 88:603-7. https://doi.org/10.1007/BF02265282

7. Messaoud O, Rekaya MB, Ouragini H, Benfadhel S, Azaiez H, Kefi R, et al (2012) Severe phenotypes in two Tunisian families with novel XPA mutations: evidence for a correlation between mutation location and disease severity. Arch Dermatol Res 304:171-6. https://doi.org/10.1007/s00403-011-1190-4

8. Amr K, Messaoud O, El Darouti M, Abdelhak S, El-Kamah G (2014) Mutational spectrum of Xeroderma pigmentosum group A in Egyptian patients. Gene 533:52-6. https://doi.org/10.1016/j.gene.2013.09.125

9. Tamhankar PM, Iyer SV, Ravindran S, Gupta N, Kabra M, Nayak C, et al (2015) Clinical profile and mutation analysis of xeroderma pigmentosum in Indian patients. Indian J Dermatol Venereol Leprol 81:16-22. https://doi.org/10.4103/0378-6323.148559

10. Maeda T, Sato K, Minami H, Taguchi H, Yoshikawa K (1994) Severe neurological abnormalities associated with a mutation in the zinc-finger domain in a group A xeroderma pigmentosum patient. Br J Dermatol 131:566-70. https://doi.org/10.1111/j.1365-2133.1994.tb08562.x

11. Nishigori C, Moriwaki S, Takebe H, Tanaka T, Imamura S (1994). Gene alterations and clinical characteristics of xeroderma pigmentosum group A patients in Japan. Arch Dermatol 130:191-7. https://doi.org/10.1001/archderm.1994.01690020057009

12. Satokata I, Tanaka K, Miura N, Narita M, Mimaki T, Satoh Y, et al (1992). Three nonsense mutations responsible for group A xeroderma pigmentosum. Mutat Res 273:193-202. https://doi.org/10.1016/0921-8777(92)90080-M

13. Kondoh M, Ueda M, Ichihashi M (1995) Correlation of the clinical manifestations and gene mutations of Japanese xeroderma pigmentosum group A patients. Br J Dermatol 133:579-85. https://doi.org/10.1111/j.1365-2133.1995.tb02709.x

14. Ghafouri-Fard S, Fardaei M, Miryounesi M (2016) A novel 5 nucleotide deletion in XPA gene is associated with severe neurological abnormalities. Gene 576:379-80. https://doi.org/10.1016/j.gene.2015.08.039

15. Satokata I, Tanaka K, Yuba S, Okada Y (1992) Identification of splicing mutations of the last nucleotides of exons, a nonsense mutation, and a missense mutation of the XPAC gene as causes of group A xeroderma pigmentosum. Mutat Res 273:203-12. https://doi.org/10.1016/0921-8777(92)90081-D

16. Kore-eda S, Tanaka T, Moriwaki S, Nishigori C, Imamura S (1992) A Case of Xeroderma Pigmentosum Group A Diagnosed With a Polymerase Chain Reaction (PCR) Technique. Arch Dermatol 128:971-4. https://doi.org/10.1001/archderm.1992.01680170103016

17. Mimaki T, Tanaka K, Okada Y, Minami R, Tachi N, Nagai A, et al (1992) Allelic heterogeneity in group A xeroderma pigmentosum. Acta Neurol Scand 85:327-30. https://doi.org/10.1111/j.1600-0404.1992.tb04052.x

18. Sun Z, Zhang J, Guo Y, Ni C, Liang J, Cheng R, et al (2015) Genotype-phenotype correlation of xeroderma pigmentosum in a Chinese Han population. Br J Dermatol 172:1096-102. https://doi.org/10.1111/bjd.13429

19. Moriwaki S, Nishigori C, Teramoto T, Tanaka T, Kore-eda S, Takabe H, et al (1993) Absence of DNA repair deficiency in the confirmed heterozygotes of xeroderma pigmentosum group A. J Invest Dermatol 101:69-72. https://doi.org/10.1111/1523-1747.ep12360046

20. Terada A, Tanizaki H, Aoshima M, Tokura Y, Moriwaki S (2017) Lichen planus-like keratosis emerging in a pediatric case of xeroderma pigmentosum group A. J Dermatol 44:e152-e153. https://doi.org/10.1111/1346-8138.13883

21. Nakao A, Tanizaki H, Yu A, Araki A, Moriwaki S (2018) Case of xeroderma pigmentosum group A with West syndrome. J Dermatol 45:e334-e336 https://doi.org/10.1111/1346-8138.14487

22. Yokota K, Sano K, Murofushi Y, Yoshimaru D, Takanashi Y (2018) Neurochemistry evaluated by MR spectroscopy in a patient with xeroderma pigmentosum group A. Brain Dev 40:931-933. https://doi.org/10.1016/j.braindev.2018.06.013

23. States JC, Myrand SP (1996) Splice site mutations in a xeroderma pigmentosum group A patient with delayed onset of neurological disease. Mutat Res 363:171-7. https://doi.org/10.1016/0921-8777(96)00004-3

24. Maeda T, Sato K, Tanaka T, Minami H, Taguchi H, Mimaki T, et al (2000). Compound heterozygous group A xeroderma pigmentosum patient with a novel mutation and an inherited reciprocal translocation. Br J Dermatol 143:174-9. https://doi.org/10.1046/j.1365-2133.2000.03611.x

25. Mimaki T, Nitta M, Saijo M, Tachi N, Minami R, Tanaka K (1996) Truncated XPA protein detected in atypical group A xeroderma pigmentosum. Acta Paediatr 85:511-3. https://doi.org/10.1111/j.1651-2227.1996.tb14074.x

26. Kondoh M, Ueda M, Nakagawa K, Ichihashi M (1994) Siblings with xeroderma pigmentosum complementation group A with different skin cancer development: importance of sun protection at an early age. J Am Acad Dermatol 31:993-6. https://doi.org/10.1016/S0190-9622(94)70270-5

27. Negishi I, Kato G, Moriwaki S, Ishikawa O (2002). Compound heterozygosity for the Xeroderma pigmentosum complementation group A gene associated with a mild phenotype. Eur J Dermatol 12:536-9.

28. Takahashi Y, Endo Y, Sugiyama Y, Inoue S, Iijima M, Tomita Y, et al (2010) XPA gene mutations resulting in subtle truncation of protein in xeroderma pigmentosum group A patients with mild skin symptoms. J Invest Dermatol 130:2481-8. https://doi.org/10.1038/jid.2010.137

29. Zhang J, Cheng R, Yu X, Sun Z, Li M, Yao Z (2017) Expansion of the genotypic and phenotypic spectrum of xeroderma pigmentosum in Chinese population. Photodermatol Photoimmunol Photomed 33:58-63. https://doi.org/10.1111/phpp.12283

30. Cleaver JE, Charles WC, Thomas GH, McDowell ML (1995) A deletion and an insertion in the alleles for the xeroderma pigmentosum (XPA) DNA-binding protein in mildly affected patients. Hum Mol Genet 4:1685-7. https://doi.org/10.1093/hmg/4.9.1685

31. Takahashi Y, Endo Y, Kusaka-Kikushima A, Nakamaura S, Nakazawa Y, Ogi T, et al (2017) An XPA gene splicing mutation resulting in trace protein expression in an elderly patient with xeroderma pigmentosum group A without neurological abnormalities. Br J Dermatol 177:253-257. https://doi.org/10.1111/bjd.15051

32. Sidwell RU, Sandison A, Wing J, Fewcett HD, Seet JE, Fisher C, et al (2006) A novel mutation in the XPA gene associated with unusually mild clinical features in a patient who developed a spindle cell melanoma. Br J Dermatol 155:81-8. https://doi.org/10.1111/j.1365-2133.2006.07272.x

33. Soufir N, Ged C, Bourillon A, Austerlitz F, Chemin C, Stary A, et al (2010) A prevalent mutation with founder effect in xeroderma pigmentosum group C from North Africa. J Invest Dermatol 130:1537-42. https://doi.org/10.1038/jid.2009.409

34. Santiago KM, França de Nóbrega A, Rocha RM, Rogatto SR, Achatz MI (2015) Xeroderma pigmentosum: low prevalence of germline XPA mutations in a Brazilian XP population. Int J Mol Sci 16:8988-96. https://doi.org/10.3390/ijms16048988

35. Salomao RPA, Pedroso JL, Barsottini OGP (2018) Neurological manifestations of xeroderma pigmentosum due to XPA gene mutation. Pract Neurol 18:489-491. https://doi.org/10.1136/practneurol-2018-001888

36. Yang Y, Ding B, Wang K, Bu D, Tu P, Zhu X (2004) DNA-based prenatal diagnosis in a Chinese family with xeroderma pigmentosum group A. Br J Dermatol 150:1190-3. https://doi.org/10.1111/j.1365-2133.2004.05938.x

37. Nasir M, Ahmad N, Sieber CM, Latif A, Malik SA, Hameed A (2013) In silico characterization of a novel pathogenic deletion mutation identified in XPA gene in a Pakistani family with severe xeroderma pigmentosum. J Biomed Sci 20:70. https://doi.org/10.1186/1423-0127-20-70

38. Messaoud O, Ben Rekaya M, Cherif W, Talmoudi F, Boussen H, Mokhtar I, et al (2010) Genetic homogeneity of mutational spectrum of group-A xeroderma pigmentosum in Tunisian patients. Int J Dermatol 49:544-8. https://doi.org/10.1111/j.1365-4632.2010.04421.x

39. Nishigori C, Zghal M, Yagi T, Imamura S, Komoun MR, Takebe H (1993) High prevalence of the point mutation in exon 6 of the xeroderma pigmentosum group A-complementing (XPAC) gene in xeroderma pigmentosum group A patients in Tunisia. Am J Hum Genet 53:1001-6.

40. Bensenouci S, Louhibi L, De Verneuil H, Mahmoudi K, Saidi-Mehtar N (2016) Diagnosis of Xeroderma Pigmentosum Groups A and C by Detection of Two Prevalent Mutations in West Algerian Population: A Rapid Genotyping Tool for the Frequent XPC Mutation c.1643_1644delTG. Biomed Res Int 2016:2180946. https://doi.org/10.1155/2016/2180946

41. Kindil Z, Senhaji MA, Bakhchane A, Charoute H, Chihab S, Nadifi S, et al (2017) Genetic investigation of XPA gene: high frequency of the c.682C>T mutation in Moroccan XP patients with moderate clinical profile. BMC Res Notes 10:704. https://doi.org/10.1186/s13104-017-3042-6

42. Messaoud O, Ben Rekaya M, Kefi R, Chebel S, Boughammoura-Bouatay A, Bel Hadj Ali H, et al (2010) Identification of primarily neurological phenotypic expression of xeroderma pigmentosum complementation group A in a Tunisian family. Br J Dermatol 162:883-6. https://doi.org/10.1111/j.1365-2133.2010.09646.x
